# The effect of a natural disaster on handgrip strength in prepubertal Indian children exposed to a severe cyclone during the prenatal and early postnatal growth

**DOI:** 10.1038/s41598-021-86845-4

**Published:** 2021-04-02

**Authors:** Sławomir Kozieł, Raja Chakraborty, Kaushik Bose, Zofia Ignasiak, Aleksandra Gomula, Natalia Nowak-Szczepanska

**Affiliations:** 1 Department of Anthropology, Ludwik Hirszfeld Institute of Immunology and Experimental Therapy, Polish Academy of Sciences, Wroclaw, Poland; 2 Department of Anthropology, Dinabandhu Mahavidyalaya, Bongaon, West Bengal India; 3 Department of Anthropology, Vidyasagar University, Midnapore, West Bengal India; 4 Department of Biostructure, University School of Physical Education in Wroclaw, Wroclaw, Poland

**Keywords:** Developmental biology, Ecology, Physiology

## Abstract

Natural disasters (NDs) experienced by women and their children during prenatal and infant growth may have long-lasting effects on offspring’s development. Handgrip strength (HGS) is one of the measures of muscular strength and an indicator of health status. This study compared HGS in children exposed to cyclone Aila in India during their prenatal and infant growth compared to a control group from a non-affected, adjacent area. The total sample involved 444 boys and 423 girls aged 7–9 years, categorised into 3 groups: prenatally exposed to Aila, exposed to Aila in infancy, and the control group, non-exposed to Aila. Results revealed that prenatally exposed children of both sexes had significantly lower HGS than the controls (at least, p < 0.001 in boys; p < 0.05 in girls). On the other hand, the postnatally exposed boys, but not the girls, showed lower HGS than the controls. A significant effect of a group factor (ND exposure) on HGS was observed even after controlling for confounding variables (age, height, BMI, birth weight, gestational age; at least, p < 0.05). Our findings indicate that prenatal or early postnatal experience of a ND may have association with impaired HGS in prepubertal children.

## Introduction

Previous studies on humans and animals have shown that stress during prenatal and early postnatal periods can seriously affect growth and development of offspring^1^. Adverse intrauterine conditions may affect fetal development leading to deterioration of childhood physical and mental health as well as subsequent health hazards^2^. Poor birth outcomes, such as, low birth weight^3^, preterm birth^4^ and still birth^5^ may occur due to prenatal maternal stress (PNMS). Several mechanisms have been suggested to interpret these effects, particularly, elevated levels of maternal glucocorticoids, which can cross the placenta or get transmitted through breastfeeding and disrupt child development^6,7^ and may also cause deregulation of the hypothalamic–pituitary–adrenal axis^8^, which is involved in metabolic pathways and likely mediates in the developmental origin of adult diseases^9,10^.

Most studies evaluate the level of stress using questionnaires or interviews, considering different stressful events during gestation^3,11^. However, researchers investigating different factors resulting into maternal stress, face challenges regarding several issues, such as, defining maternal stress, evaluation of subjective stress, etc.^12^ Besides, methodological problems are inherent in studies on the effects of PNMS, with regard to controlling for gestational timing and other confounding risk factors^13,14^. The ethical issues also delimit the scope of random assignment of stressors to human subjects, particularly, pregnant mothers, to conduct experimental studies. A natural disaster (ND), however, creates a distinctive state of affair that provides a wider scope of study of PNMS and its after effects with higher relative objectivity.

A ND, such as earthquake or super cyclone, acts as a discrete and sudden stressor that quasi-randomly distributes hardship and stress to a population, and thereby exposing a large number of pregnant women at varying gestational stages. The challenges of life during, and after a disaster, are assumingly more uniform if there is less heterogeneity in people’s living conditions, such as, in remote rural areas. Therefore, a research design that uses a severe ND as a potential stressor acting on a whole community at a narrow time frame, irrespective of several different factors which may affect PNMS, is a relatively unique method in this kind of studies^15,16^.

The first elaborate research project that used ND to investigate the impact of PNMS on child development was carried out following the 1998 Quebec Ice Storm in Canada^12,17,18^. Similar other disaster research included the Iowa Flood (2008) Study in USA^15^ and the Queensland Flood (2011) Study in Australia^19^. Results from these studies suggested that a ND had an independent effect on PNMS causing long-lasting biological effects in offspring, including epigenetic imprints that persisted into late childhood and early adolescence^16,20,21^.

Exposure to a ND during pregnancy was also shown to influence birth outcomes, such as gestation length, birth weight, birth length, head circumference, and growth ratios of the offspring, independent of other potential factors^20^. However, whether the exposure to maternal stress during prenatal or neonatal stage is associated with impairment of functional parameters, such as handgrip strength (HGS), has not yet been systematically addressed^22^. The measurement of HGS is a relatively simple, rapid and inexpensive method to assess upper extremity strength in clinical and population studies^23,24^. It reflects muscular strength in children, adolescents and young adults^25^. HGS is a reliable indicator of skeletal muscle function in the general population^26^, as well as overall health status^27^. HGS is positively associated with lean muscle mass^28^, nutritional status^29,30^, and negatively with morbidity and mortality^31^. It may also predict survival in serious systemic diseases^30^. Higher HGS was also associated with reduced cardio-metabolic risk factors in adolescents^32^ and low HGS in adolescence was associated with increased risk of premature mortality. The magnitude of the association was similar to established risk factors, such as elevated blood pressure and body mass index (BMI)^33^. HGS was also shown to be inversely associated with socioeconomic status in children and adolescents^34^. However, to our knowledge, there is no study till date investigating association of HGS during childhood with prenatal and/or early postnatal exposure to a severe ND.

On 25 May 2009, a tropical cyclone *Aila*, classified as ‘severe cyclonic storm', hit India and Bangladesh at a speed of 120–140 km per hour and devastated the coastal islands of Sunderbans^35^. It claimed 138 human lives, killed numerous cattle and destroyed human properties^36,37^. Thus far, scientific studies conducted to portray the aftermath of the cyclone have mostly dealt with ecological^35,38^ and economical effects^39^, livelihood and resilience^40^, post disaster health hazards like diarrhea and cholera outbreaks^41–43^ and the psychological impacts on the adult population^44^. However, according to our knowledge, no study, so far, considered growth, development and physical characteristics of children exposed to the PNMS and early postnatal distress due to *Aila*.

Consequently, the objective of our study was to assess the difference in HGS, as an indicator of muscular strength and function, between three groups of children, viz., (i) who faced in-utero PNMS, (ii) who faced the post-disaster hardship in the early postnatal period (infancy) and (iii) who did not experience PNMS, as their pregnant mothers lived in similar and neighbouring areas that were not affected by *Aila*. It was hypothesised that children who experienced a ND in utero or during infancy, revealed poorer parameters of muscular functions development, as reflected in HGS, compared to the children not affected by the calamity.

## Methods

### Study design and participants

The present cross-sectional study, as a part of a larger research project investigating the long-term effect of cyclone *Aila* on the growth and development of children, adopted a quasi-experimental retrospective design that used three cohorts of children—two experimental groups and one control group. Experimental cohorts of children were recruited from the two Islands of the Sunderban delta region, where the effect of the super cyclone was the severest and human lives were acutely threatened due to their disconnectedness with the mainland district^40,45^. Children from the first group were intrauterine on the day of the cyclone and born between June 2009 and February 2010. Based on the previously mentioned devastating effects of the cyclone, it could be speculated that the pregnant women experienced substantial trauma amid the massive destruction and threat to their future existence during the cyclone. The second group of children was also recruited from the same islands where the first experimental cohort was included. However, they were born over less than/nearly two years preceding *Aila* and faced all the post-disaster perils during their infancy. As discussed elsewhere in this article, the post-disaster condition of the islands was uninhabitable and full of unforeseen hardship, including adverse epidemiological conditions. Therefore, it was obvious that the early postnatal phase of development of those children born before *Aila*, was also harsh and with considerable developmental insults. Finally, the control group belonged to the same birth cohort as the first group of children who were prenatally affected by Aila, but were recruited from the villages of the adjacent district, North 24 Parganas, that did not face the cyclone. The people of this area did not differ from the Sunderban people in respect of their origin, culture and language. Several demographic and socio-economic data were also incorporated in the study design to ensure that the populations from which all study groups were recruited were similar in other aspects, except only that the control group did not face the cyclone. The other parameters were also statistically controlled, as required in analyses.

### Study areas and settings

Sunderban is the world’s largest single mangrove area and a UNESCO global heritage site, spreading through the extreme south of the eastern India and, also, Bangladesh. Indian Sunderban includes approximately over a hundred small and large islands, out of which, 54 are inhabited by nearly 4 million people. The rest is protected forest, known as the Sunderban Tiger Reserve^46,47^. The islanders survive on rain-fed, mostly, mono-crop agriculture, forest products and fishing from rivers and estuaries. A large proportion of people belong to the scheduled castes and tribes, who are, in general, marginalized communities. Approximately 50% of the farmers are landless labourers^47,48^.

The two experimental groups of children were recruited from two islands, Satjelia and Kumirmari. These were among the last inhabitable islands of Indian Sundarbans, communicable only by waterways, and also the most destroyed by *Aila*^49^. Data were collected from 22 primary schools in Satjelia Island and 13 schools in Kumirmari Island. The data on postnatally *Aila*-exposed children were collected from the same schools at Satjelia. The control children came from 21 primary schools in villages located in the neighbouring district, North 24 Parganas, under the community development block, namely, Bongaon. These areas did not experience *Aila*, except a mild storm normal for the season, and without flooding. The distance of these villages from the sub-district headquarter town, Bongaon, is approximately 15–20 km. All children under this project were studied at their respective primary schools.

### Sampling

A total of 987 children were initially identified. Their guardians were sent formal request to participate in this study. Nine hundred and thirty one children were present in school during the study visits while 56 children were absent from school due to unknown reasons. Four children were found to be sick, and thus, not measured. In total, 927 children participated in the study. Therefore, the approximate response rate was 94%. However, important information for questionnaire was not available from the parents of 37 children even after completion of measurements. Data on another 22 children were excluded as their dates of birth were found to be incorrectly and falling out of the target range. Overall, data on 868 children were included in the final database of the project, where informed consent from the mothers/legal guardians were available.

Based on the variables required, the present study included 837 children (426 boys and 411 girls) representing the three study groups: (i) children whose exposition to Aila occurred prenatally but the aftereffect of the disaster lasted postnatally (prenatal stress group) (175 boys and 161 girls), (ii) children exposed to Aila during their infancy (early postnatal stress group) (109 boys and 107 girls), and (iii) children non-exposed to Aila (control group) (142 boys and 143 girls). The study followed a multi-stage sampling strategy. The first two groups lived in the two islands which were selected after consulting published information as well as through focused observation, interview and group discussions with local people. These islands were the most affected ones in terms of devastation, the level of rise of water level relative to human habitations, the loss of properties, agricultural lands and the time taken for restoration to moderate living conditions and economy.

All children, fulfilling the criteria of the research design, studying in primary schools, were potential participants. Each school was requested to participate and identify students who were born between June 2009 and February 2010 and/or between March 2007 and May 2009. Schools were visited on a mutually agreed prefixed date when the presence of all students and their mothers was ensured by the school authority. Trained field investigators collected data along with measurements. Children with symptoms of illness or any abnormality were not included. Children or guardians who disagreed to participate after knowing the study’s purpose were also exempted.

### Research ethics

The Institutional Ethics Committee for Research on Human Subjects, West Bengal State University, India, approved the project (approval no. WBSU/IEC/14/03, dated 13.11.2017). The Protection of Children from Sexual Offences (POCSO) Act of India was also respected. All methods were carried out in accordance with relevant guidelines and regulations. Informed consent was obtained from all mother participants, and for children from parent and/or legal guardian.

### Measurements

HGS was measured using an isometric dynamometer (Saehan Corporation, South Korea) following standard procedure^22,24^. The instrument was adjusted according to hand size. The children were asked to stand with hands freely hanging vertically along the lateral side of body. Each child was instructed to hold the instrument in the correct manner and to exert as much pressure as possible. A second round of measurements was performed after an interval of two minutes. The maximum value of the two measurements for each hand was then recorded. Information on hand dominance of children was confirmed by their mothers and recorded accordingly during data collection. During the study, we found that reported left handedness was extremely low for the whole sample (~ 3.4%, 29 children). Therefore, we did not consider hand dominance in statistical analyses. It is known that, in general, left handedness remains less than 10% in populations^50,51^. In a study among Indian schoolchildren, left handedness was found to be 3.2% and 4.2%, in two samples of 4–11 and 6–11 years of age, respectively^52^. Therefore, our finding of only 3.4% of left handedness was not unusual or significantly influencing our study outcome. The average HGS value of both hands was considered to be the principal outcome variable as an estimate of overall HGS, irrespective of dominant hands. Body height and weight were measured following standard techniques^50,53^ with anthropometer (GPM, Switzerland) and weight scale (Libra), respectively. The gestation period and birth weight were recalled by the mothers and recorded accordingly.

### Statistics

Differences between the three groups were assessed by one-way analysis of variance (ANOVA) for normally distributed dependent variable. Two-way ANOVA was applied to assess differences in HGSs, where group and sex were factors. Differences between groups within each sex were assessed by post hoc Tukey’s HSD test for unequal sample sizes. Effects of independent variables, including the group factor, were assessed by multiple analysis of covariance (ANCOVA) in generalised linear model (GLM) with logit link function. In the model, parameters of HGS of right and left hands were dependent variables, while the group factor and sex were independent factors. The following variables were included in a model as the confounding variables: age, height, BMI, gestational age, and birth weight. The second order interaction effect between groups and sex was additionally included in the model. Significance of the effect in this model was assessed by Wald’s chi-square test. It is worth mentioning that, for the ANCOVA, values for all variables were available for 599 children (300 boys; 299 girls). All calculations were performed using Statistica 13.1 software.

## Results

Table 1 presents the descriptive statistics of the variables included in the study. However, information on birth weight and the gestational age, was not available for all individuals. In boys and girls, all three groups showed significant differences in age, height, BMI and gestational age. The control group showed higher birth weight and BMI. The children from the postnatal stress group were the tallest, most likely due to being older than children from the other two groups.

**Table 1:** Descriptive statistics of age, birth weight, gestational age, height and BMI by experimental groups and controls in boys and girls. Differences in groups were assessed by one-way analysis of variance.

Groups	Boys			Girls		
	N	Mean	SD	N	Mean	SD
**Age (years)**						
Prenatal stress	175	8.06	0.21	161	8.08	0.24
Postnatal stress	109	9.31	0.50	107	9.28	0.36
Control	142	8.35	0.23	143	8.48	0.23
	F = 543.39; p < 0.001			F = 647.85; p < 0.001		
**Birth weight (g)**						
Prenatal stress	138	2692	551	123	2628	492
Postnatal stress	75	2726	554	75	2630	387
Control	112	2757	529	123	2743	561
	F = 0.43; n.s			F = 1.98; n.s		
**Gestational age (week)**						
Prenatal stress	160	37.89	2.35	151	38.09	2.30
Postnatal stress	74	37.66	2.44	74	37.50	2.96
Control	128	39.06	1.84	129	38.53	3.29
	F = 13.48; p < 0.001			F = 3.11; p < 0.05		
**Height (cm)**						
Prenatal stress	175	120.55	5.21	161	120.78	4.90
Postnatal stress	109	127.94	5.94	107	126.82	6.39
Control	142	123.31	5.46	143	122.36	5.40
	F = 60.95; p < 0.001			F = 39.96; p < 0.001		
**BMI (kg/m**^**2**^**)**						
Prenatal stress	175	14.43	1.45	161	14.34	1.74
Postnatal stress	109	14.57	1.67	107	14.34	1.58
Control	142	16.16	2.19	143	16.48	2.66
	F = 41.72; p < 0.001			F = 49.93; p < 0.001		

**Figure 1:**
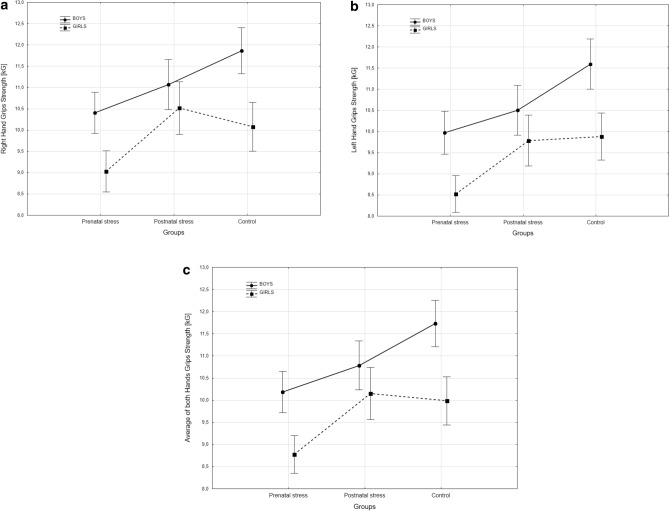
(**a**,**b**) Weighted marginal means of right (**a**), left (**b**) and average (**c**) HGS in boys and girls by experimental groups and controls.

Descriptive statistics of HGS of right and left hands, as well as the average HGS of both hands among boys and girls in three groups and the results of two-way analysis of variance are presented in Table 2. There was no significant difference in HGS between the Aila exposed children from the two islands under study (p > 0.05). Also, there were significant effects of groups, sex and second order interactions in right-, left- and average HGS. Among the boys, the prenatally *Aila* exposed group showed significantly lower values for right and left hands as well as in the average of HGS of both hands than the controls (post hoc comparison: p < 0.001 for all HGS), whereas the postnatally exposed group showed lower values in left hand and average HGS, but not in right hand HGS, than the controls. However, both the exposed groups did not significantly differ in their HGS of either hand or the average of both hands. On the other hand, similarly to the boys, the prenatally *Aila* exposed girls showed significantly lower HGS than the control groups (post hoc comparisons: p < 0.05, 0.001 and 0.01, respectively, for right, left and average HGS). However, the girls in the postnatally Aila exposed group did not differ significantly from the controls (Fig. 1). It is noteworthy, that unlike in boys, the prenatally exposed girls had significantly lower values of all measures of HGS than the postnatally exposed girls as well (p < 0.01).

**Table 2:** Means (SD) HGS of both hands and the differences assessed by two-way analysis of variance according to group and sex.

Groups	Boys				Girls				
	N		Mean	SD	N		Mean		SD
	**Right hand HGS (kg)**								
1. Prenatal stress	167	10.36		2.66	154			9.12	2.69
2. Postnatal stress	109	10.03		2.37	107			10.59	2.52
3. Control	142	11.96		2.75	142			10.13	3.18
				F	p				
		Group		19.46	< 0.001				
		Sex		36.55	< 0.001				
		Group × Sex		4.02	< 0.05				
Post-hoc p-values	1 vs. 2–n.s.; 1 vs. 3–< 0.001 2 vs. 3–n.s				1 vs. 2–< 0.01; 1 vs. 3–< 0.05 2 vs. 3–n.s				
	**Left hand HGS (kg)**								
1. Prenatal stress	166	9.85		2.86	154			8.57	2.36
2. Postnatal stress	109	10.43		2.39	107			9.91	2.52
3. Control	142	11.79		3.05	143			9.89	3.10
				F	p				
		Group		26.33	< 0.001				
		Sex		39.50	< 0.001				
		Group × Sex		3.72	< 0.05				
Post-hoc p-values	1 vs. 2–n.s.; 1 vs. 3–< 0.001 2 vs. 3–< 0.01				1 vs. 2–< 0.01; 1 vs. 3–< 0.001 2 vs. 3–n.s				
	**Average of both hands HGS (kg)**								
1. Prenatal stress	166	10.10		2.63	154			8.85	2.37
2. Postnatal stress	109	10.73		2.22	107			10.25	2.42
3. Control	142	11.86		2.69	142			10.01	3.03
				F	p				
		Group		25.13	< 0.001				
		Sex		42.17	< 0.001				
		Group × Sex		4.29	< 0.05				
Post-hoc p-values	1 vs. 2–n.s.; 1 vs. 3–< 0.001 2 vs. 3–< 0.05				1 vs. 2–< 0.01; 1 vs. 3–< 0.01 2 vs. 3–n.s

Results of multivariate analysis of covariance including the effects of second order interactions between groups and sex are shown in Table 3. Controlling for the confounding factors, pre- and postnatal stress conditions, compared to the control situation (without *Aila*), showed a significant impact (group effect) on HGS of both hands (Wald’s χ^2^ right: 8.32, p < 0.05 and left: 13.94, p < 0.001) in both sexes. This is in conformity with the descriptive results shown in Table 2. Nevertheless, it is worth mentioning that we tested if there was any difference due to the exclusion of children for missing data required for ANCOVA. There was no significant difference in HGS between children included and not-included in the analyses (Boys: N = 300 vs. 117; right hand t = 0.06 p = 0.9509; left hand t = 0.23 p = 0.8159; average t = 0.09 p = 0.9247. Girls: N = 299 vs. 105; right hand t = 1.20 p = 0.2298; left hand t = 0.69 p = 0.4933; average t = 0.99 p = 0.3221; results not shown).

**Table 3:** Results of the analysis of covariance by GLM, where HGS was dependent variable.

	Right hand (N = 599)		Left hand (N = 599)		Average (N = 599)	
	Wald’s χ^2^ (df)	p	Wald’s χ^2^ (df)	p	Wald’s χ^2^ (df)	p
Group	7.99 (2)	< 0.05	14.36 (2)	< 0.001	12.54 (2)	< 0.01
Sex	23.81 (1)	< 0.001	27.27 (1)	< 0.001	28.83 (1)	< 0.001
Age	0.26 (1)	n.s	1.49 (1)	n.s	0.83 (1)	n.s
Height	58.47 (1)	< 0.001	69.51 (1)	< 0.001	67.12 (1)	< 0.001
BMI	4.29 (1)	< 0.05	6.49 (1)	< 0.01	6.05 (1)	< 0.05
Birth weight	1.17 (1)	n.s	0.92 (1)	n.s	1.19 (1)	n.s
Gestational age	1.64 (1)	n.s	1.55 (1)	n.s	1.85 (1)	n.s
Group × sex	7.49 (2)	< 0.05	4.60 (2)	n.s	6.74 (2)	< 0.05

## Discussion

The present study investigated a probable effect of prenatal-, and/or early postnatal exposure to a ND on the neuromuscular development in prepubertal children (7–9 years age), reflected in HGS. As the other ND projects, the Aila study in India also provided an opportunity to examine the plausible role of PNMS, associated with the exposure to a ND, on children’s physical outcomes. This was possible due to the randomness and severity of the cyclone on pregnant or newly mothers in the affected areas, irrespective of their natural differences in age, body size and birth parameters. The results of the present study revealed that the boys exposed to *Aila*, both prenatally and postnatally, showed significantly reduced HGS compared to the control group, except for the right hand HGS, in which the postnatal group did not differ with the control. However, the pre- and postnatally exposed boys did not significantly differ between them in any of the three HGS measures. The prenatal stress caused by *Aila* seemed to have had different effects on HGS in boys and girls. In boys, both the disaster exposed groups (prenatal and early postnatal) showed significant differences compared with controls (except for right hand), whereas, in girls, the prenatally *Aila* exposed group consistently showed significantly lower HGS, in both hands and average, compared to both the postnatally *Aila* exposed and control groups. The postnatally exposed girls, contrary to boys, did not differ significantly from the control group. The reason of this sexual dimorphism could not be ascertained in this study. Still these results are not accidental and seem to be reliable as they were found with the use of appropriate statistical methods that controlled possible confounding factors. The reasons for the obtained sex differences may have been influenced by differential ecosensitivity and environmental variation in phenotypic traits according to sex, which may also depend on specific outcome being studied^54^. However, a recent international twin study revealed that genetics, rather than common environmental factors, plays an increasingly important role in the variation in body size from early childhood to late adolescence, among boys, but not in girls, who demonstrated a relatively greater impact of environment^55^. HGS, nevertheless, is linked not only with body size, but also with the whole neuromuscular development. In another recent study, exposure to tobacco smoke during prenatal or early postnatal periods affected HGS measures in boys, but not in girls^22^. Particularly, previous research on prenatal maternal stress caused by a ND (The Queensland Flood Study) also proposed that the male fetuses are less protected against prenatal maternal stress, since subjective PNMS is associated with lower levels of expression of the glucocorticoid-promoting gene found only in boys^19^. However, based on the results, it seems that exposure to a ND during the crucial prenatal stage of development, affected both sexes in terms of the effect of ND on HGS. At the same time, early postnatal exposure to a ND was more detrimental for boys, due to their higher ecosensitivity to this environmental factor, compared to girls, who might have had better coping mechanism to adverse external conditions in their early postnatal life. However, these are only logical assumptions of probable reasons, and it should be noted that higher ecosensitivity of a particular sex may depend on the outcome being studied. Further in-depth comparative studies on other functional and health indicators among these cohorts might lead to more assenting conclusions.

As stated earlier, the most effective measure of HGS was based on the average value the two hands’ measurements. Thus, in conformity with the hypothesis, the pre- and postnatally exposed children, irrespective of sex, showed significantly lower average HGS than the non-exposed control children. Additionally, the prenatally Aila-experienced children of both sexes, showed significantly lower values than the controls in all the three HGS measures, and in girls, the prenatal group also showed consistently lower values compared to both the other two groups. HGS is a reliable indicator of musculoskeletal growth and development^25^, and the prenatal period has a profound effect on these processes. Although the major growth of volume and size of muscle occurs postnatally, the development of muscles begins during 6–8 weeks of gestation when the primary muscle fibres appear followed by the secondary fibres at 8–18 weeks. Thus, the number of fibres is almost fixed at birth^56,58^. This number may be modified by intrauterine nutrition^57^. Placental insufficiency may also have adverse effect on growth of skeletal muscle in foetus^58^. Studies using animal models showed that the skeletal muscle weight could be reduced disproportionately to body weight due to placental insufficiency^59^. Human foetus’s affected by intrauterine growth retardation (IUGR) often demonstrate deficient skeletal muscle mass^60^. Stressful events during the later stage of pregnancy have been negatively linked with offspring motor development^61^. Behaviours causing physiological stress, such as smoking (by pregnant mothers), may also exposed offspring to a higher risk of IUGR and low birth weight^62^, as well as lower HGS^22^. Nonetheless, it should be noted that in our study sample, the potential confounder of smoking was absent since the women from the study regions were predominantly non-smokers due to cultural reasons. The present study also took an account of birth weight as it is a potential determinant of HGS^63,64^ through improved prenatal growth of muscle tissue and/or postnatal development of the musculoskeletal system^22^. The Quebec Ice Storm study showed that PNMS due to the disaster independently affected birth outcomes^20^. Lower HGS in stress-affected children in the present study was independent of birth weight. Another plausible source of variation in HGS could be age. Our study groups consisted of a rather narrow age range (7–9 years) and within the context of children’s development, it was the same prepubertal period of growth. However, since there were some significant differences in age between the study groups, we included age in subsequent statistical analyses, which showed that its effect on HGS was non-significant. This confirmed our assumption that in our study groups, this age range was relatively narrow to influence the findings significantly.

ND is believed to impose short, medium and long-term hardships. It exerts an immediate blow to household assets, income and livelihood streams, physical infrastructure and common property resources^65,66^, and further disadvantages people at risk from human resources in the long term. HGS does not only indicate the qualitative and functional aspects of muscle strength, but also predicts nutritional and general health status^30,67^. Thus, the present study, like the similar previous ones, indicated a long term biological impact on growth and development in children. Decreased HGS among the ND exposed children might have been indicative of their depleted musculoskeletal health status, having serious health implications in their later adulthood.

The present study also allowed us to understand the enduring effects of mothers’ stress during pregnancy on children’s postnatal development. It is, perhaps, the first of its kind on ND-induced maternal stress affecting child growth and development in a developing country, where living conditions are harsh and emergency services are not proficient. However, it was also suggested that the impact of ND can vary systematically across socio-economic groups^68^. In relation to the present study, the mothers were from a homogeneous rural population, with a very low socioeconomic variation, were highly dependent on the natural environment in which they lived, and their lifestyle was also not very divergent. Another important advantage of our study was a large sample size compared to similar previous studies that included a relatively small number of subjects. These studies had also been conducted in economically developed countries with efficient rescue services. Despite direct danger of serious injury and even death, there were no reports of death directly caused by the above-mentioned NDs. In contrast, the magnitude of damage of the tropical cyclone, Aila, which was responsible for 138 reported deaths, was much greater than that caused by the Quebec ice storm or Queensland and Iowa floods^44^.

This study had some limitations. Firstly, birth weight and gestational length were based on maternal recall. In a number of cases, this information could not be verified from official records, such as birth certificates, which were not available from the mothers or were lost during cyclone *Aila*. Secondly, we did not consider the difference in subjective distress between mothers due to the ND. However, these differences were expected to have been adjusted due to the quite large sample size and the relative uniformity of living conditions and island environment. Previously similar studies on the effects of ND had attempted to correlate specific objective experiences during ND with later biological outcomes in children. Our study did not include such individual measures of specific objective hardships. Although, the present study had similar objectives, it followed a different design by using control groups, unlike previous studies. Those studies investigated the intra group variation among the exposed population in terms of association between nature- and degrees of hardships and their effects (outcomes). In contrast, our study examined the comparison between exposed- and non-exposed groups as a whole. The study also lacked physiological measures of stress, such as cortisol level, associated with stressful life-events and psychopathology^68^. Moreover, it can be argued that the differences in HGS between islands and the control group might have been due to general differences between mainland and island children. However, there is no research indicating such differences in HGS. Thus, it is rather a speculative assumption. It may also be noted that in our results the postnatally exposed girls (from islands) did not differ significantly from the control group (from mainland). Therefore, potential differences in HGS due to differences between islands and mainland seemed to have been negligible. Nevertheless, another limitation of this study involves a lack of normative data with standardized values of HGS from the studied region; however, the aim of this investigation was a comparison of the effect of a natural disaster on exposed *versus* non-exposed children from the adjacent, neighbouring region, similar in living conditions; thus, potential data standardization did not seem to be necessary.

Although the present study did not objectively confirm that all mothers and children experienced the same level of hardships, it was evident from the available reports that the cyclone Aila affected all members of community at the same time with similar destructive force, independent of individual differences in socio-economic conditions or other objective stresses which were unrelated to the occurrence of the ND. Individual experiences were very similar, if not the same. Since the study was conducted some years after the disaster, we could not rely on data on the objective stresses of the mothers’ which had occurred just before and/or during the time of the disaster, based on their memories. Therefore, we considered Aila as an agglomeration of distinct environmental stressors. Moreover, another logical assumption was that the effect of Aila did not vary to a large extent among the families since the people in the study areas were not highly varied in socio-economic and other living conditions. Nevertheless, we drew our assumptions regarding socioeconomic and psychological hardships of the studied groups from previous studies. Our hypothesis was, thus, scientifically precise within the ambit of its specific limitations. It is a matter of further investigation to ascertain the extent of psycho-biological stressors and the ways in which individuals implement coping mechanisms and also the genetic endowment that could contribute to such intra-population variation.

In conclusion, the findings indicated that the prenatal and early postnatal exposure to a severe natural disaster seemed to have had a negative effect on the development of HGS in children aged 7–9 years. Given the recent increase in the frequency and intensity of natural disasters due to climate change^69^, a greater understanding of the impacts of in-utero exposure to additional ND induces PNMS on optimal child development is essential. The exact mechanism by which such stress acts on the placenta and affects fetal programming requires further investigation. Moreover, it should be of particular concern to implement emergency interventions, especially, to the biological conditions of a child, in populations affected by natural disasters in developing countries with insufficient resources.
